# Polymorphous acral eruption and Covid-19

**DOI:** 10.11604/pamj.supp.2020.35.2.23576

**Published:** 2020-05-26

**Authors:** Youness Elkhachine, Abdessamad Sakkah, Ihssane Hallab, Abderrazak Jakar, Mohamed Elhaouri, Jalal Elbenaye

**Affiliations:** 1 Department of Dermatology, Moulay Ismail Military Hospital, Meknes, Morocco; 2 Faculty of Medicine and Pharmacy, Sidi Mohamed Ben Abdellah University, 30000, Fes, Morocco

**Keywords:** Covid-19, skin, acral eruption, polymorphous, young, immunological

## Abstract

Clinical manifestations associated with covid-19 seem to be not limited to the lungs and widen to affect several organs including skin. Through two cases well documented, we report cutaneous manifestations strongly suggestive of Covid-19 which dermatologists are beginning to report worldwide and which we proposed to call them polymorphous acral eruption.

## Commentary

**Figure 1:**
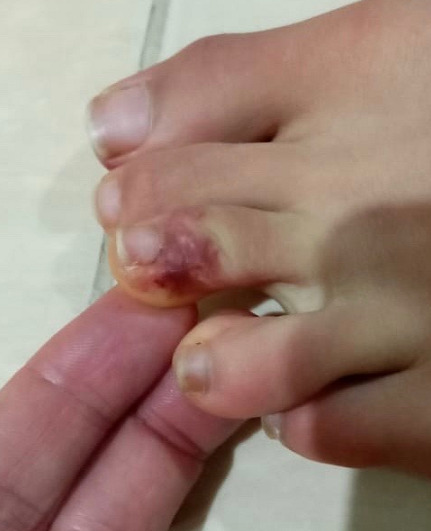
Erythematopurpuric lesions of the toes and soles “chilblains like”

**Case 1:** a 17-year-old adolescent with no medical history, especially chilblains, had asymptomatic erythematopurpuric lesions of the toes and soles for four days (Figure 1). There was neither fever nor respiratory distress but a mild cough. He had been complaining of headache and sore throat for the past eight days. He had contact with a family member with covid-19 positive. There were no biological abnormalities including lymphopenia, thrombocytopenia, hypercoagulability except a slight increase of inflammatory markers. A chest CT showed peripheral ground-glass opacities in both lungs. The Sars-cov-19 RT-PCR was negative. These findings suggest a liability Covid-19 especially as there was no evidence for another etiology.

**Figure 2:**
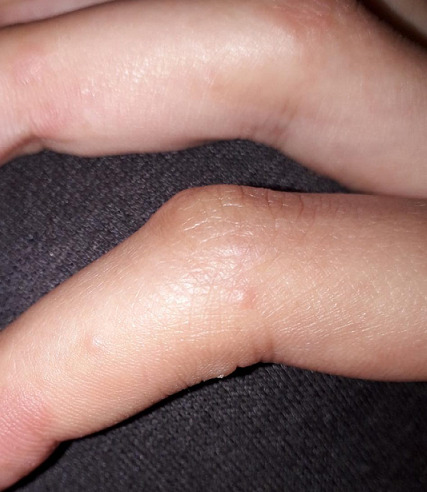
Non-itching painless papulovesicular lesions of the fingers

**Case 2:** a 23-year-old young woman, with no pathological history, presented for a week papulovesicular lesions of the fingers without pain or pruritus (Figure 2), concomitant with anosmia. She complained of severe asthenia, sore throat, dry cough and headache for ten days without fever. There was mild lymphopenia and moderate inflammatory syndrome. Sars-cov-19 RT-PCR was negative but rapid serological test showed Covid-19 IgG positive.

Since description of Covid-19, we are witnessing every day new clinical observations and new affected organs; skin is one of them, despite Darlenski and Tsankov ruling out a cutaneous manifestation of covid-19 [1]. Indeed, cutaneous manifestations related to sars-cov-19 were first mentioned by Italian authors. It was mainly erythematous rash, widespread urticaria and a case of chickenpox-like vesicles. Trunk was the main involved region. Apparently there was not any correlation with disease´s severity [2]. Hoenig LJ and al have reported a patient with presumptive diagnosis of COVID-19 infection presenting an asymptomatic erythematous, slightly edematous facial eruption [3].

Curiously, several dermatologists have noticed acral eruptions like chilblains [4], purpura ischemic lesions or even vesicles, occurring mainly in adolescents and young people with suspected Covid-19 but no finding could establish a direct link with sars-cov-19. They called them acrosyndromes linked to Covid-19. Our two cases corroborate these findings. Polymorphous clinical presentation (papulovesicles, purpura, urticaria, chilblains) with characteristic acral distribution suggested that it would be linked to an immunological mechanism affecting the small vessels rather than to a direct effect of the virus, especially as these lesions occurred a few days after the onset of symptoms. This was observed in a covid-19 patient with chilblains whose histological findings showed a lichenoid lymphocytic infiltrate without thrombosis [4]. Qualifying these lesions as acrosyndromes could be confusing and above all does not cover all the clinical forms that may be encountered in Covid-19. So, in our opinion, it seems more judicious to call them polymorphous acral eruption. Further studies would be needed to confirm these findings and would open the way to an understanding of the Covid-19 pathogenesis as well as proposing therapeutic applications.

## Competing interests

The authors declare no competing interests.
